# TruthTrust: Truth Inference-Based Trust Management Mechanism on a Crowdsourcing Platform

**DOI:** 10.3390/s21082578

**Published:** 2021-04-07

**Authors:** Jiyuan Zhou, Xing Jin, Lanping Yu, Limin Xue, Yizhi Ren

**Affiliations:** 1 School of Cyberspace, Hangzhou Dianzi University, Hangzhou 310018, China; 181270009@hdu.edu.cn (J.Z.); jinxing@hdu.edu.cn (X.J.); 202270024@hdu.edu.cn (L.X.); renyz@hdu.edu.cn (Y.R.); 2 School of Information Engineering, Hangzhou Dianzi University, Hangzhou 310018, China

**Keywords:** crowdsourcing, trust model, collusion requester, spam worker, truth inference

## Abstract

On a crowdsourcing platform, in order to cheat for rewards or sabotage the crowdsourcing processes, spam workers may submit numerous erroneous answers to the tasks published by requesters. This type of behavior extremely reduces the completion rate of tasks and the enthusiasm of honest users, which may lead a crowdsourcing platform to a failure. Defending against malicious attacks is an important issue in crowdsourcing, which has been extensively addressed by existing methods, e.g., verification-based defense mechanisms, data analysis solutions, trust-based defense models, and workers’ properties matching mechanisms. However, verification-based defense mechanisms will consume a lot of resources, and data analysis solutions cannot motivate workers to provide high-quality services. Trust-based defense models and workers’ properties matching mechanisms cannot guarantee the authenticity of information when collusion requesters publish shadow tasks to help malicious workers get more participation opportunities. To defend such collusion attacks in crowdsourcing platforms, we propose a new defense model named TruthTrust. Firstly, we define a complete life cycle system that from users’ interaction to workers’ recommendation, and separately define the trust value of each worker and the credence of each requester. Secondly, in order to ensure the authenticity of the information, we establish a trust model based on the CRH framework. The calculated truth value and weight are used to define the global properties of workers and requesters. Moreover, we propose a reverse mechanism to improve the resistance under attacks. Finally, extensive experiments demonstrate that TruthTrust significantly outperforms the state-of-the-art approaches in terms of effective task completion rate.

## 1. Introduction

In the last decade, an increasing number of businesses have transformed their business models by going online [1]. In recent years, the process of transformation has accelerated and businesses have entered into the cloud computing era, where virtually turning everything as a service (XaaS) [2] has become a distinct possibility [3]. A notable example is the emergence of crowdsourcing platforms. Some well-known crowdsourcing platforms, such as Wikipedia (https://www.wikipedia.org, accessed on 1 April 2021), FreeLancer (https://www.freelancer.com, accessed on 1 April 2021) and Amazon Mechanical Turk (https://www.mturk.com, accessed on 1 April 2021), have proved that the wisdom of crowds [4] possesses tremendous potential in addressing complex problems [5]. On a crowdsourcing platform, a crowdsourcing process starts when a requester publishes Human Intelligence Tasks (HITs) and workers who satisfy the requirements can submit their answers according to the task descriptions [6].

Such a crowdsourcing process is extremely vulnerable to spam workers who want to get a profit from cheating since they can easily get permission from participating in tasks [4]. More seriously, the collusion strategy is widely used in malicious users, known as Rank Boosting [7], where dishonest workers boost their overall trust levels by participating in easy tasks or in fake tasks published by themselves or conspiracy requesters. Furthermore, in the case that more than 50% of the workers are malicious, spam workers can manipulate specific answers to destroy the system [8]. The extensive existence of spam workers will greatly reduce the reliability of crowdsourcing platforms and the enthusiasm of honest participants, which will lead a crowdsourcing platform to a failure [9]. Thus, the issues of how to establish trust ratings and choose trustworthy workers become prominent.

A well-known attack launched by spam workers has happened on the Waze [10], the most popular crowdsourced map service, which offers users more ways to actively share information on accidents, landmarks, local fuel prices, etc. [10]. However, in recent years, people adopt collude strategies such as simulating a traffic jam by not moving to decrease the efficiency of the platform [11]. Due to the existence of attacks, there is a lot of misinformation on Waze. The fake information resulted in urban road congestion and reduced the enthusiasm of normal users to provide right answers. In fact, there are few effective tools today to identify whether the origin of traffic requests are real mobile devices or software scripts [12].

In practice, spam workers can take strategies to masquerade themselves as trustworthy participants, which make them extremely hard to be detected [13], and it can often be difficult to identify whether the requester who posts the task is a normal user or plays a role conspiring with malicious workers [14]. In general, according to whether to provide effective services or feedback, workers and requesters can be generally classified into three strategies:

- S1: Honest Strategy. Workers with honest strategies always provide good services, and requesters give objective and real evaluations over all workers.
- S2: Collusive Strategy. Spam workers through collusive requesters always get high ratings to make themselves disguised as normal workers. When the collective workers are chosen to complete a HIT published by a normal requester, they always provide invalid services. On the other hand, requesters with a collusive strategy only recruit spam workers.
- S3: Camouflage Strategy. When the workers with camouflage strategy are chosen to complete a HIT published by an honest requester, they will provide valid services with a certain probability. On the other hand, the requester with the S3 strategy will also recruit some honest workers to complete HITs and give them real evaluations with a certain probability.

With the strategies S2–S3, spam workers may acquire a “good” reputation and then continuously pose threats to crowdsourcing platforms. According to previous research, it can be divided into four categories to improve the service quality: (1) Verification-based Defense Model [15,16,17]; (2) Data Analysis Solution [18,19,20,21]; (3) Workers’ Properties Matching Mechanism [22,23,24,25]; and (4) Trust-based Model [4,26,27,28].Verification-based Defense Models adopt the method of re-checking the submitted answers to discover spam workers so that these models require a lot of resources. Data Analysis Solutions filter out valuable services by analyzing data submitted by workers. This approach is limited to mobile crowdsourcing platforms and cannot motivate workers to provide high-quality services. Workers’ Properties Matching Mechanisms usually establish match models to distribute tasks to workers. However, the attributes of the workers are submitted by workers or requesters. These mechanisms cannot guarantee the authenticity of workers’ properties. Trust-based Models select workers by establishing trust evaluations. However, existing trust models rarely consider the impact of collusion attacks, resulting in the effectiveness of these models decreasing significantly.

Therefore, ensuring the authenticity of the information about workers and requesters in the crowd has become the main challenge. In order to solve this problem, we use the truth inference algorithm [29] which can find the truth value from various information sources. Truth inference is widely used in big data and social media analysis where noisy information is inevitable [30]. Specifically, our model is established based on a simple and efficient CRH [31] algorithm which can obtain the closest real information by assigning weights and minimizing the loss function in the information set.

In this paper, we focus on colluding attacks and mixed camouflage strategies attacks and propose a new trust model TruthTrust based on the CRH algorithm. Furthermore, in order to motivate workers to provide high-quality services, we present a three-layer complete life cycle model in which past behaviors can also affect the current trust evaluation. We separately define the trust evaluation of workers and the reliability of requesters to distinguish malicious users more effectively. Furthermore, we design a reverse mechanism to solve the problem that the CRH algorithm is invalid under specific attacks. Finally, during the experiment, we compare our model with different existing crowdsourcing trust management models and analyze its effectiveness. The main contributions of this paper are listed as follows:

(1) We propose TruthTrust, a complete life cycle system that separately considers the trust evaluation of workers and the reliability of requesters to resist colluding and mixed camouflage strategy attacks.

(2) We establish a reverse mechanism to counter special attack scenarios, and define the global trust vector to improve the anti-attack performance as well as motivate workers to provide high-quality services.

(3) We implement adequate experiments to simulate malicious strategies S2–S3 and compare the performance of TruthTrust with many existing trust management models. Our results illustrate that the proposed TruthTrust model could provide accurate and reliable results in the presence of different attacks.

The rest of the paper is organized as follows: Section 2 presents related work about improving the quality of service in crowdsourcing platforms. In Section 3, we introduce our TruthTrust model in detail. In Section 4, we conduct simulation experiments to compare with existing models and evaluate the effectiveness of our model. In the last section, we conclude our work and outline future research directions.

## 2. Related Work

In order to improve the service quality of the crowdsourcing platform and reduce the attacks of malicious workers, previous studies have mainly made the following solutions.

### 2.1. Verification-Based Defense Model

In general, verification-based defense models utilize testing questions to estimate whether a worker is honest or not and organizes reviewers to re-check the submitted answers to discover spam workers. In particular, Kittur et al. [15] pointed out that publishing tasks with preset testing tasks (i.e., the tasks with known answers) together can effectively differentiate the quality of different workers in a task. Chen et al. [16] designed a verification mechanism that regards workers who always give consistent answers as trustworthy workers. Correspondingly, the workers who commonly submit contradictory answers are marked as suspiciously spam workers. Hirth et al. [17] proposed two mechanisms (MD and CG) to detect the cheating behaviors of workers. They proposed that it is ineffective or costly to apply verification problems and manual re-checking simultaneously. In general, verification-based defense models may identify malicious workers with high accuracy. However, such a type of model cannot prevent spam workers from participating in tasks beforehand and may consume a large number of extra resources to verify the quality of all workers.

### 2.2. Data Analysis Solution

The solution of data analysis usually focuses on the data analysis phase by using data processing methods such as Bayesian [18], machine learning clustering algorithm [19], and truth inference [20,21], in order to find the low quality of service. Lin et al. [20] proposed a Sybil-resistant truth inference framework for MCS, which included three account grouping methods in pair with a truth inference algorithm to defend against the Sybil attacks. Liu et al. [19] proposed a probabilistic model to jointly infer multi-dimensional trust of workers, multi-domain properties of questions, and true labels of questions. Du et al. [18] proposed a novel Bayesian co-clustering truth inference model, which produced an estimation while taking into account the entity clusters and the user clusters for the task of observation aggregation. Huang et al. [21] investigated the data poisoning attacks on truth inference and proposed an approach against such attacks through additional source estimation and source filtering before data aggregation.

This type of solution has good timeliness and has no need for a priori knowledge. However, if the system is under Sybil attacks, the effectiveness of these solutions will be significantly reduced. At the same time, data analysis solutions cannot motivate workers to provide high-quality services.

### 2.3. Workers’ Properties Matching Mechanism

Workers’ properties matching mechanisms are often matched and optimized choices between workers and tasks based on previous worker records and task requirements. Ye et al. [22] proposed a context-aware trust model which consists of two types of context-aware trust: task type based trust (TaTrust) and reward amount based trust (RaTrust). Moreover, they regarded the issue of model trustworthy worker selection as a multi-objective combinatorial optimization problem, which is NP-hard. Yuen et al. [23] proposed a task matching idea that utilized the past preference of a worker and a task to produce a list of available tasks in the order of best matching with the worker during its task selection stage. Jiang et al. [24] proposed an approach for context-aware reliable crowdsourcing in social networks. This approach decomposed complex tasks according to social attributes while taking into account the social reputation of workers. Karger et al. [25] proposed a method to learn the crowdsourcing outcomes iteratively for assigning tasks to the appropriate workers. Workers’ properties matching mechanisms can match more suitable tasks according to workers’ preferences and improve the efficiency of crowdsourcing. However, such models can not guarantee the authenticity of the property of the workers and may be vulnerable to attack when workers counterfeit fake records.

### 2.4. Trust-Based Model

Trust-based models usually build trust evaluation models based on the performance of workers or feedback from requesters, regard the staff with a lower trust level as spam workers, and then prohibit such spam workers from participating in any task. Yu et al. [26] proposed a recommendation model based on the similarity of workers and requesters. Kantarci et al. [27] proposed a collaborative trustworthiness approach for mobile crowdsensing, which leverages the naive centralized reputation value by incorporating statistical and vote-based trust scores that take advantage of social network theory. Ye et al. [4] proposed a new Trust Sub-network Extraction algorithm (TSE) to discover more requesters who can provide opinions for generating recommendations. Ye et al. [28] proposed a trust vector-based threat defense model CrowdDefense. They established a Crowdsourcing Trust Network (CTN) composed of requesters, workers, and their transaction relations, and analyzed three threat patterns of spam workers. Moreover, they assumed that there exist requesters with different identities, and part of them is trustworthy.

Trust-based models can motivate workers to provide high-quality services, and the computational overhead of other solutions is relatively low. However, existing trust models rarely consider the scenario where the requesters in the crowd collude with malicious workers, which may lead to a significant reduction in the effectiveness of current trust-based models.

## 3. TruthTrust Model

**Figure 1:**
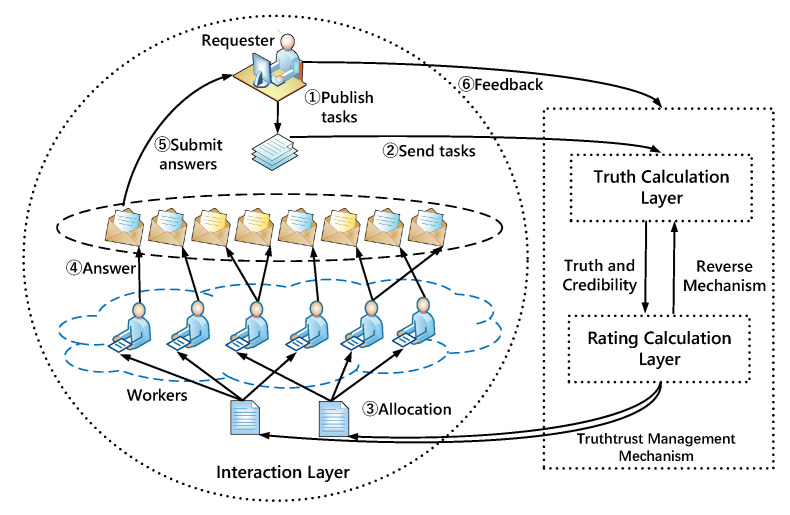
TruthTrust System.

In this section, we clearly illustrate the design of TruthTrust. As shown in Figure 1, TruthTrust is a complete life cycle and divided into three layers: Interaction Layer, Truth Calculation Layer, and Rating Calculation Layer. The Interaction Layer mainly illustrates the interaction and attack model of workers and requesters using S2 and S3 strategies. In the Truth Calculation Layer, we propose a feedback calculation model based on a truth inference algorithm. Specifically, we set up a reverse mechanism to defend threat patterns that the truth inference framework is invalid. In the Rating Calculation Layer, we define a global trust vector of workers and requesters based on truth and weight calculated by the Truth Calculation Layer, and then recommend workers according to the global trust.

### 3.1. Interaction Layer

A typical crowdsourcing process can be decomposed into four procedures: (1) Requester publishing tasks; (2) Task assignment; (3) Worker submit answers; and (4) Feedback and reward. At the beginning of a crowdsourcing process, a requester publishes tasks to all the workers in the form of an open call [32]—then according to trust evaluation or worker preferences to decide who can submit the answer to the task. Afterward, if a worker’s answer is approved in a task, the worker will be granted the reward of the task according to the reward mechanism deployed [33]. In the end, requesters can rate the staff after the transaction is completed. For example, each time requester *i* gets a service from worker *j*, it may rate the transaction as satisfaction $tr_{\left(i,j\right)}=1$ or dissatisfaction $tr_{\left(i,j\right)}=0$. In the Truth Calculation Layer, we will collect feedback information to calculate the credibility of requester *i*, and the service quality of worker *j*.

**Figure 2:**
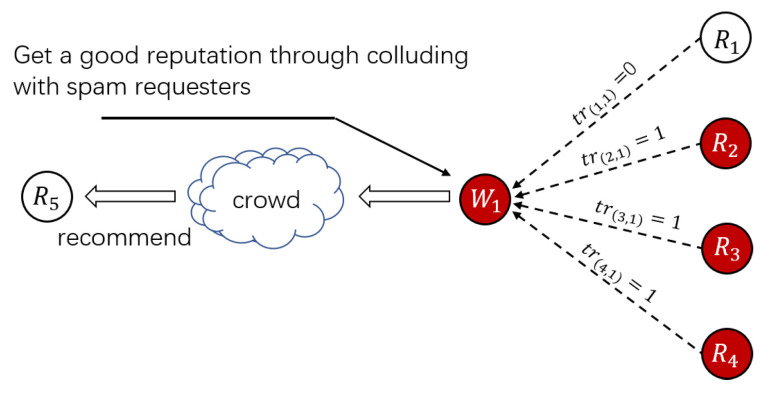
Threat Pattern A.

In the actual crowdsourcing process, with the aim to get rewards easily, spam workers will improve their reputations by adopting strategies S2–S3 through participating in shadow HITs constantly. The answer of shadow HIT is preset and shown to spam workers in advance. In shadow HITs, malicious participants can be separately classified into spam requester, camouflage requester, and camouflage worker. Typically based on different participants, threat patterns can be divided into three categories:

Threat Pattern A (Spam Workers Collude with Spam Requesters): Spam workers ceaselessly trade with spam requesters in shadow HITs to obtain a high trust level. For example, as shown in Figure 2, a spam worker $W_{1}$ deploys reputation by obtaining satisfactory feedback from spam requesters $R_{2}-R_{4}$ in shadow HITs. In contrast, honest workers have difficulty participating in shadow HITs published by spam requesters $R_{2}-R_{4}$. Once $W_{1}$ takes part in a tremendous amount of shadow HITs, $W_{1}$ will get a “good” reputation and some worker recommendations or worker selection mechanisms will consider $W_{1}$ as a trustworthy worker. If only one-sided worker reputation is considered, honest requesters $R_{5}$ may take $W_{1}$ as an honest worker [28]. When $W_{1}$ participates in a normal HIT, $W_{1}$ will submit erroneous answers to destroy the crowd system.

**Figure 3:**
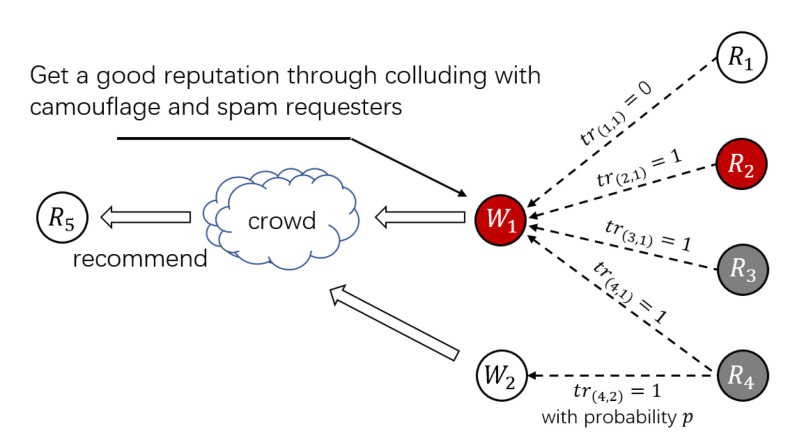
Threat Pattern B.

Threat Pattern B (Spam Workers Collude with Spam and Camouflage Requesters): In Threat Pattern A, it is easy for malicious workers and requesters to form a small group, which is easily detected by clustering methods. In order to enhance the camouflage ability of malicious users, camouflage requesters are considered in Threat Pattern B where spam workers transact with both spam requesters and camouflage requesters in shadow HITs to improve their reputations. Camouflage requesters publish both normal HITs and shadow HITs and provide a true evaluation of the honest workers with probability *p*. For example, as shown in Figure 3, spam worker $W_{1}$ boots its reputation by shadow HITs published by camouflage requesters $R_{3}$ and $R_{4}$ and spam requester $R_{2}$. At the same time, a certain number of normal tasks will be published for honest workers such as $W_{2}$ to complete. After the tasks are completed, honest workers will be truly evaluated with probability *p*. With the assistance of camouflage requesters, malicious users can avoid forming a small bloc that can be detected easily. In addition, the reputation of the honest workers will also be affected by camouflage requesters.

**Figure 4:**
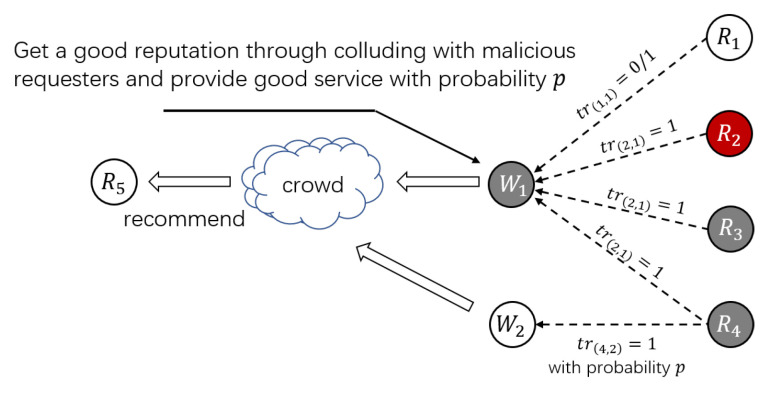
Threat Pattern C.

Threat Pattern C (Camouflage Workers Collude with Spam and Camouflage Requesters): In Threat Pattern B, we only consider the camouflage strategy of the requester, similarly, malicious workers will also adopt the camouflage strategy in order not to be detected easily. In Threat Pattern C, camouflage workers collude with both spam requesters and camouflage requesters, which can conceal the malicious workers deeply. Camouflage workers may provide some normal services, which makes them similar to normal workers. Figure 4 shows an instance of Threat Pattern C in which camouflage worker $W_{1}$ gains a good reputation from spam requester $R_{2}$ and camouflage requesters $R_{3}$ and $R_{4}$. At the same time, $W_{1}$ will complete some normal tasks such as the tasks issued by $R_{1}$, and get some good feedback, making it hard to detect. In the meantime, when camouflage workers are regarded as honest workers, they will submit the same answer or erroneous answers with the probability 1−p to destroy the system.

The above threat patterns make malicious workers difficult to be detected. In order to improve the anti-attack capability of the crowdsourcing platform and improve the service quality of workers, we should analyze the real information in the feedback from heterogeneous requesters. In the Truth Calculation Layer, the truth about the service quality *V* of workers and the credibility *C* of requesters can be calculated to select trustworthy workers and resist threat patterns A–C. Next, we will introduce our calculation model in detail.

### 3.2. Truth Calculation Layer

Considering the above-mentioned attack scenarios, there must be much fake feedback from malicious requesters. How to filter out real and effective feedback is the main problem we need to solve. The truth inference algorithm can calculate the closest information to the real information through the optimization model. Thus, we utilize the CRH algorithm that is simple and widely used to process feedback [30]. In the Truth Calculation Layer, in order to distinguish the trust attributes of workers and requesters, we can calculate the credibility of requesters and the truth of worker’s service quality.

The general principle of truth discovery works as follows: If a source provides trustworthy information frequently, it will be assigned a high reliability; meanwhile, if one piece of information is supported by sources with high reliabilities, it will have more of a chance to be selected as truth. In our model, the truth value represents the service qualities of workers analyzed from the feedback information, and the weight value represents the credibility of the feedback information provided by the requesters. Based on the above principle, we utilize the following optimization framework to deal with heterogeneous feedback in this process:

(1) $$\begin{array}{c} \underset{\vartheta^{*},W}{min}\;f\left(\vartheta^{*},W\right)=\sum_{k=1}^{K}\omega_{k}\sum_{i=1}^{N}d\left(v_{i}^{*},tr_{\left(k,i\right)}\right)\\ s.t.\;\delta (W)=1,\;W\in S \end{array}$$

where $v_{i}^{*}$ denotes the truth of service quality about worker *i*. The truths of all workers about the different service qualities are saved in a truth table $\vartheta^{*}$, where the *i*-th term is $v_{i}^{*}$. Requester’s credibility is denoted as $W=\{\omega_{1},\omega_{2},...,\omega_{k}\}$ in which $\omega_{k}$ is the reliability degree of the *k*-th requester. A higher value of $\omega_{k}$ indicates that the *k*-th requester is more reliable and the feedback from this requester is more likely to be accurate.

By minimizing the objective function $f(\vartheta^{*},W)$, we can search about the values of two groups of unknown variables $\vartheta^{*}$ and W, which corresponds to the truths set and the requester weight set, respectively. In this framework, to figure out Equation (1), there are two types of functions that need to be optimized:

- Loss function. *d* is a loss function defined based on the satisfaction of service. This function measures the difference between the feedback $tr_{\left(k,i\right)}$ and the identified truth $v_{i}^{*}$. When the feedback approaches the true value, the loss function should output a low value, and when the feedback deviates from the truth, the loss function should output a high value.
- Regularization function. δ(ω) represents the constraint of the requester’s credibility. If each requester’s credibility $\omega_{k}$ is unconstrained, then the $\omega_{k}$ can be simply taken as −∞, resulting in the unbounded optimization problem. To constrain the requester credibility ω into a certain range, we need to specify the regularization function δ(ω) and the domain *S* that is the entire requester set. For simplicity, we set the value of δ(ω) to be 1.

In order to minimize the objective function in Equation (1), first, we need to get the initial truth value $\vartheta^{*}$ by voting or averaging from the results of feedback tables. Then, we perform the following two steps iteratively:

Step1: Weights Update. First, we estimate the truth $\vartheta^{*}$ and weigh each requester according to the difference between the feedback and the truth. At this step, we fix the values of the truth and compute the weight that jointly minimizes the objective function subject to the regularization constraints. In order to distinguish the weight of the requesters more clearly, the following regularization function is used to get more contribution from the truth obtained by reliable requesters:

(2) $$\delta \left(\omega \right)=\sum_{k=1}^{K}exp\left(-\omega_{k}\right)$$

This function regularizes $\omega_{k}$ by limiting the sum of exp$\left(-\omega_{k}\right)$. To expand the difference of requesters’ weights, the negative logarithmic function maps the weights in the range of 0 and 1 to the range of 0 and *∞*.

Assuming that the truth values are fixed and with constraint Equations (1) and (2), the optimization problem is convex. Therefore, the weight calculation can be consistent with the distance between $tr_{\left(k,i\right)}$ and $v_{i}^{*}$. The global optimal solution is as follows:

(3) $$\omega_{k}=-log\left(\frac{\sum_{i=1}^{N}d\left(v_{i}^{*},tr_{\left(k,i\right)}\right)}{\sum_{k^{\prime }=1}^{K}\sum_{i=1}^{N}d\left(v_{i}^{*},tr_{\left(k^{\prime },i\right)}\right)}\right)$$

where *K* is the set of requesters that provide feedback to the same workers with requester *k*, and *N* is the set of workers evaluated by requester *k*.

Step2: Truths Update. We update the truth of each item in order to minimize the difference between the feedback and the truth since the weight of each requester $\omega_{k}$ is fixed. The feedback $tr_{\left(k,i\right)}$ made in the Interaction Layer is type (0,1), so we use the (0,1) loss function in which an error is occurred, if we use the (0,1) loss function, an error will occur if the feedback is not the same as the truth, thus we define the deviation from the true value $v_{i}^{*}$ to the observed value $tr_{\left(k,i\right)}$ as follows:

(4) $$d\left(v_{i}^{*},tr_{\left(k,i\right)}\right)=\left\{\begin{array}{cc} 1 & if\;tr_{\left(k,i\right)}\neq v_{i}^{*}\\ 0 & otherwise \end{array}\right.$$

At this step, assuming that the credibility is fixed, in order to minimize the objective function, on the *i*-th object, we set the truth as the value that gets the highest credit vote of all the possible values based on the 0–1 loss function:

(5) $$v_{i}^{*}\leftarrow \underset{v}{argmax}\sum_{k=1}^{K}\omega_{k}\cdot I\left(v,tr_{\left(k,i\right)}\right)$$

where I(x,y)=1 if x=y, and 0 otherwise.

We combine Equation (4) with the objective function in Equation (1) as follows:

(6) $$v_{i}^{*}\leftarrow \underset{v}{argmax}\sum_{k=1}^{K}\omega_{k}\cdot d\left(v,tr_{\left(k,i\right)}\right)$$

which is equivalent to Equation (5). The truth inference framework is shown in Algorithm 1.
**Algorithm 1:** Truth Inference Framework
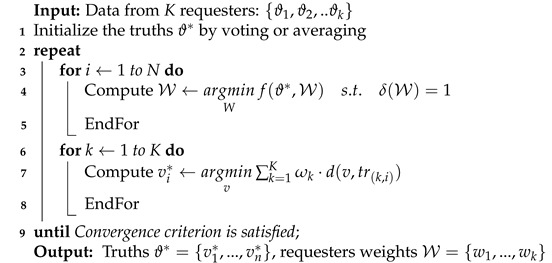


#### 3.2.1. Reverse Mechanism

In the Truth Calculation Layer, we introduce the truth inference framework to resolve conflicts from different identities of requesters. However, the basic trust inference model may be weak against attacks in specific attack scenarios, and then malicious participants may be assigned with a high trust level.

**Figure 5:**
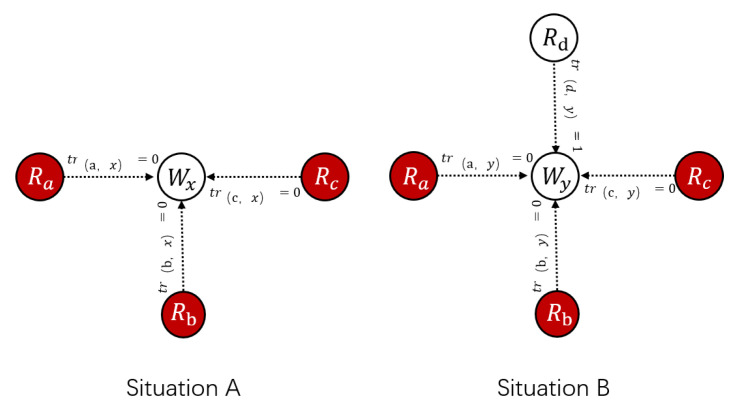
Attack situations where the CRH is invalid.

As shown in Figure 5, we exemplify two attack scenarios to illustrate the vulnerability of truth inference. In the situation *A*, after a round of transactions, worker *x* with good performance receives feedback from malicious requester a,b, and *c*. The feedback about *x* is {a:0,b:0,c:0}, which means that they are all unsatisfied with the service of worker *x*. If the CRH algorithm is adopted, the truth value of *x* will be set to 0. Then, when calculating the weights according to Equation (3), because requesters a,b,c provide feedback close to the truth, they will be assigned with high weights, which means they are credible. In addition, in situation *B*, worker *y* with good performance receives feedback from malicious requester a,b,c, and *d* where a,b,c are malicious requesters and provide fake feedback as {a:0,b:0,c:0,d:1} in which *d* is honest and provides real feedback. When malicious feedback constitutes the majority, it will also encounter the same issue in the process of initiating the truth, and, according to the optimization model a,b and *c*, will be assigned with a high weight, ultimately leading to biased results. The aforementioned situation greatly reduces the performance of calculating the truth. Thus, with the aim to reduce the impact of above situations, we propose a reverse mechanism to enhance the anti-attack ability of the system.

In order to improve the service quality of users, we establish a reverse mechanism based on the global credibility of requester termed as *C*, which has been defined in the Rating Calculation Layer to correct the truth and redistribute weights of requesters (see in Section 3.3). First, we define $\bar{\omega }$ as the baseline of all requesters:

(7) $$\bar{\omega }=\frac{\sum_{i}^{K}C_{i}}{K}\;$$

After the conclusion of the truth, we can get a set of requesters $S_{i}=\left\{R_{n},...R_{m}\right\}$ whose feedback to the worker *i* is the same as the truth $v_{i}^{*}$. Then, we define the credibility of the feedback information for worker *i* as $\gamma_{i}$:

(8) $$\gamma_{i}=\frac{\sum_{j\in S_{i}}C_{j}}{\hbar }$$

where *ℏ* is the number of requesters in the set $S_{i}$, $C_{j}$ is the global credibility of the requesters in the set $S_{i}$.

We make corrections based on the $\gamma_{i}$ after the completion of truth calculation. The true value $v_{i}^{*}$ will be reversed in the event of $\gamma_{i}$ is less than the average value $\bar{\omega }$:

(9) $$v_{i}^{+}=\left\{\begin{array}{cc} \;\bar{v_{i}^{*}} & if\;\gamma_{i}<\bar{\omega }\\ \;v_{i}^{*} & otherwise \end{array}\right.$$

**Figure 6:**
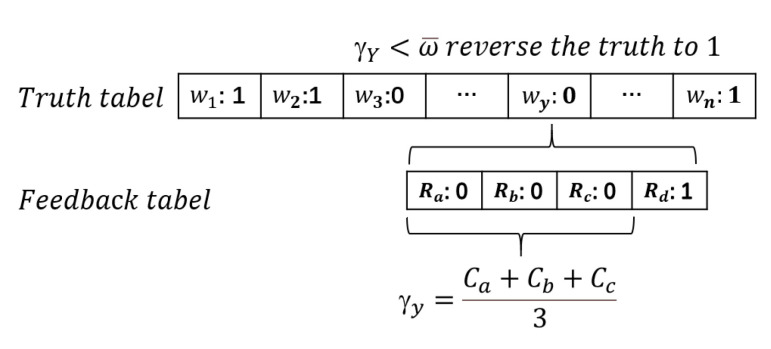
Reverse mechanism applied in situation B.

As shown in Figure 6, we will explain the principle of the reverse mechanism in the situation B seen in Figure 5. First, according to the initialized truth, the truth value of $w_{y}$ is 0. Then, we get the set of requesters $S=\{R_{a},R_{b},R_{c}\}$ with the same feedback. Then, we calculate the credibility $\gamma_{i}$, and compare it with the mean value $\bar{\omega }$. If $\gamma_{i}$ is less than the mean value $\bar{\omega }$, the truth value will be reversed to 1.

After every worker is reversely verified, we will get a new truth table $\vartheta^{+}$. Then, the weights of all requesters should be updated according to $f(\vartheta^{+},W)$, which is equivalent to Equation (3), the requesters’ weights are updated as follows:

(10) $$\omega_{k}=-log\left(\frac{\sum_{i=1}^{N}d\left(v_{i}^{+},tr_{\left(k,i\right)}\right)}{\sum_{k^{\prime }=1}^{K}\sum_{i=1}^{N}d\left(v_{i}^{+},tr_{\left(k^{\prime },i\right)}\right)}\right)$$

where $v_{i}^{+}$ is the truth about the service quality of worker *i* after reversed in from truth table $\vartheta^{+}$. According to the reverse mechanism, the assigned weight of malicious requesters can also be effectively reduced.

### 3.3. Rating Calculation Layer

In real crowdsourcing scenarios, the process of interacting is circular. However, previous truth inference algorithms or frameworks did not consider the circular feature, so, in order to capture the dynamic behavior of workers and requesters, we introduce the global trust vector of them in the Rating Calculation Layer.

In the Truth Calculation Layer, the truth $\vartheta^{+}$ and the credibility weight W can be calculated by the optimization framework. According to each optimized result $\vartheta^{+}$ and W, we define $V^{t}$ as the global trust vector of all workers and $C^{t}$ as the global credibility vector of all requesters at the time of round *t*.

$V^{t}$ is calculated from the truth $\vartheta^{+}$, and we use a method to calculate the specific properties of all workers. The method is shown as follows:

(11) $$V^{t}=V^{t-1}*\left(1-\alpha \right)+\vartheta^{+}*\alpha$$

We use the same method to calculate the credibility vector $C^{t}$ of all requesters. The difference is that we need to normalize the credibility of requesters because ω has no boundaries. Each weight $\omega_{i}$ in W will divided by $\omega_{max}$, which is the maximum value in W. Then, the calculation of $C^{t}$ is shown as follows:

(12) $$C^{t}=C^{t-1}*\left(1-\beta \right)+W*\beta$$

where α and β are parameters between 0 and 1, which can be regarded as time factors. When the value of α and β are close to 1, the global variables $V^{t}$ and $C^{t}$ will be more dependent on the latest results.

By establishing and maintaining global vectors from the Rating Calculation Layer, it enables requesters to select the right workers to complete their HITs by utilizing the trust rating *V*. With the aim to (1) ensure workers with higher trust ratings to complete the tasks, and (2) prevent the highest worker from completing the tasks all the time and letting workers with relatively high ratings have the opportunity to participate in the task, we use a probabilistic algorithm to allocate tasks. Specifically, the recommendation probability of worker *i* can be computed by:

(13) $$P_{i}=V_{i}/\sum_{r\in Q}V_{r}$$

where $V_{i}$ represents the global trust of worker *i*, and *Q* represents the set of workers that meet the task requirements. The main algorithm of TruthTrust is shown in Algorithm 2 and the validity of TruthTrust will be demonstrated in the following experiments.
**Algorithm 2:** TruthTrust Algorithm
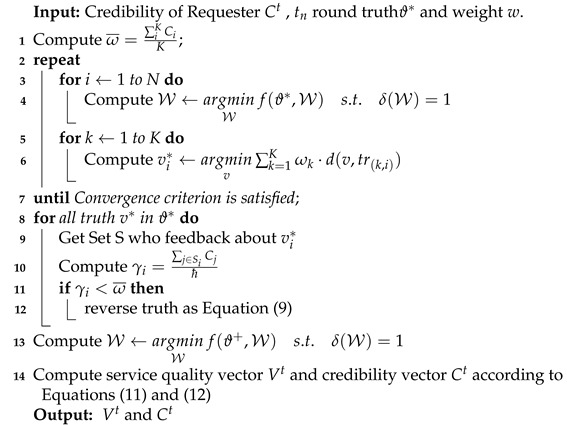


## 4. Experiments and Analysis

In this section, we have implemented simulations to illustrate the performance of the TruthTrust system. Since there is no real data about collision attacks [34], we simulated the HIT processes and different attack scenarios by establishing a simulation crowdsourcing platform. We first describe the settings of our experiment in Section 4.1, and in Section 4.2, Section 4.3 and Section 4.4, we have conducted three experiments to examine the performance of our proposed method.

### 4.1. Experimental Setup

As shown in Table 1, we initiate 1000 workers, 30 types of tasks, and 80 requesters. First, requesters publish different types of tasks that obey the standard normal distribution. Then, the truth value is calculated according to the feedback information. At the start of our experiments, each worker has the same opportunity to participate in HITs.

According to strategies S1–S3, we simulate different attack scenarios and compare the performance of defending attacks with baseline models. The specific strategic behaviors of different identities are shown in Table 2.

Based on baseline methods in our experiments, we evaluate the performance of TruthTrust and compare it with three state-of-the-art approaches CRH [31], Trust-Based Service Recommendation(TSR) [35], and AMT [36], respectively.

- CRH: The unimproved truth discovery and source reliability framework, which directly calculates the truth from the feedback data to the select appropriate workers.
- TSR: A trust-aware and resemblance based collaborative filtering recommendation model. In the TSR model, we select workers according to the similarity between workers and between requesters.
- AMT: An answer overall approval rate based trust model that is applied in the Amazon Mechanical Turk where requesters choose workers with a higher approval rate as the service provider.

### 4.2. Experiment 1: Effectiveness Analysis

This experiment aims to investigate the effectiveness of TruthTrust under different proportions of malicious requesters. We set the fraction of effective HITs as the experimental indicator which represents the percentage of normal HITs completed by effective services. By adjusting the proportion (10–50%) of malicious requester SR and CAR, and we fix the proportion of spam workers in our experiment to 25%. Along with interaction progresses, we observe TruthTrust’s performance.

**Figure 7:**
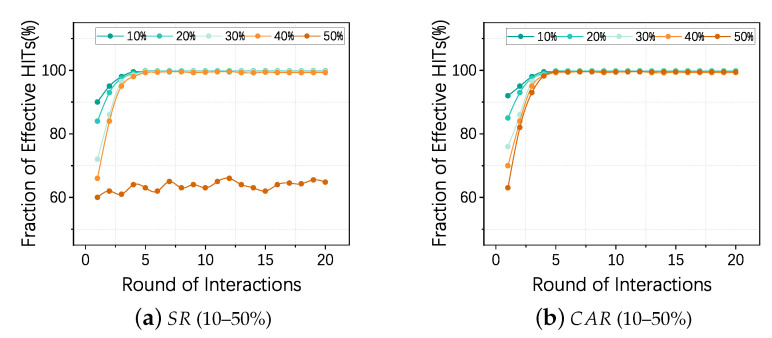
(**a**) shows the effectiveness of TruthTrust model under different ratios of SR (10–50%); (**b**) shows the effectiveness of TruthTrust model under different ratios of CAR (10–50%) and camouflage probability p=0.5.

From Figure 7a,b, we can observe: (1) In the case of different ratios of SR and CAR (10–40%) with the increase of iterations, the effective rate of HITs achieved by TruthTrust tends to converge to 99.5% and 99.8%, respectively; (2) In the extreme case SR=50%, because most of feedback in the system is fake, the truth inference framework cannot initiate the truth correctly, resulting in a significant reduction in the effectiveness of the model; (3) In the case of CAR=50% and p=0.5, most of the feedback in the system are still authentic, so the truth inference framework maintains a good performance.

### 4.3. Experiment 2: Comparative Experiment

In this experiment, we analyze the effectiveness of TruthTrust by comparing with CRH, TSR, and AMT models to investigate whether TruthTrust can effectively select honest workers when workers and requesters take strategies S1, S2, and S3. According to the different proportions of malicious members, we divide malicious attacks into three threat patterns (A–C) which are shown in Table 3. In Threat Pattern A, we only set SW & SR and adjust the proportion of SW & SR from 10% to 50%. In Threat Pattern B, we subjoin CAR to requesters and divide the proportions of SR and CAR, which is arranged from 5% to 25%. In Threat Pattern C, we subjoin CAW to workers and divide the proportions of SW and CAW. In the above threat patterns, we set the camouflage probability *p* of CAW and CAR as 0.5.

**Figure 8:**
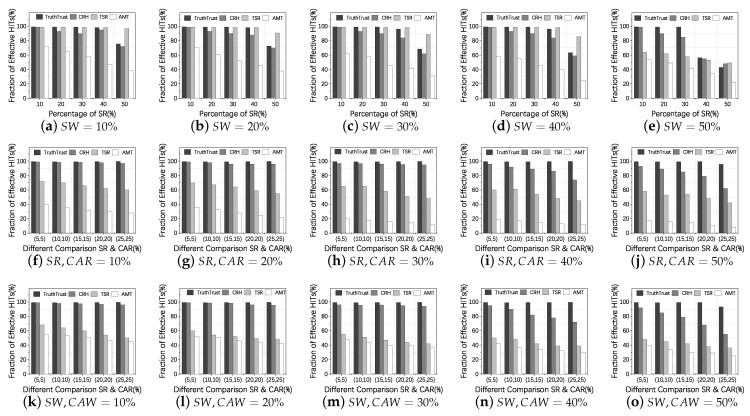
(**a**–**e**) show the effectiveness of different models under Threat Pattern A; (**f**–**j**) show the effectiveness of different models under Threat Pattern B; (**k**–**o**) show the effectiveness of different models under Threat Pattern C.

From Figure 8a–e, we can observe that, under the Threat Pattern A: (1) In the case of different proportions of SW (10–50%) and with the proportion of SR being below 30%, the averaged effective rate of TruthTrust tends to converge to 99.4%; (2) When the malicious requester tends to 50%, the effective rate of TruthTrust will be reduced because the malicious evaluation is in the majority, which makes the CRH algorithm unable to initialize the truth normally; (3) The average effective rate of the TSR model which is based on the similarity of requesters and workers tends to 99.2% when malicious participants are less than 50%. The reason is that the malicious collective and normal users have very little similarity without camouflage. However, when the proportion of malicious users exceeds 50%, the effectiveness of the TSR model is also significantly reduced. It is worth mentioning that the number of malicious requesters in the actual crowdsourcing platform would not exceed 50%; otherwise, it will cause crowdsourcing to a failure [9].

Figure 8g–j shows that under the Threat Pattern B: (1) When CAR are considered, TruthTrust always achieves the best performance in terms of selecting trustworthy workers in all the cases. On average, the proportion of effective HITs is the highest, which is 99.1%, 7.18% higher than CRH, 38.17% higher than TSR and 68.72% higher than AMT, respectively. Since most of feedback in the system is real, the truth inference framework can obtain the correct results according to the optimization model. (2) The effectiveness of the TSR model is significantly reduced, and the average effective rate is 57.6%. Because malicious requesters use a camouflage strategy, TSR is unable to effectively distinguish malicious users based on the similarity. (3) The efficiency of the AMT model which selects workers based on satisfaction is also reduced because malicious requesters not only publish shadow HITs but also give normal workers malicious feedback.

Figure 8k–o shows the ratio of effective HITs under the Threat Pattern C, and we can find: (1) Compared with other models, TruthTrust still has the best performance. On average, its effectiveness is 6.35% higher than the CRH method, 42.56% higher than the TSR method, and 61.14% higher than the AMT method. Since the reversed truth inference framework can effectively calculate the real information in heterogeneous feedback, (2) the effectiveness of the TSR model is further reduced by 16.8% on average compared to Threat Pattern B. Because the worker’s camouflage strategy affects the similarity-based clustering, which reduces the effectiveness of TSR, (3) the performance of AMT has a little improvement because, even if malicious workers participate in normal tasks, they will provide some good services because of the camouflage strategy.

In summary, TruthTrust has a relatively stable performance in Threat Patterns A-C. However, when the proportion of malicious feedback in the system is more than 50%, the effectiveness of TruthTrust will be significantly reduced. In our future research, we will try to use machine learning clustering methods [37,38] to reduce the weight of magnanimous malicious requesters. Secondly, the effectiveness of the CRH model is inferior to TruthTrust because some special attack scenarios mentioned in Section 3.2.1 reduce the effectiveness of the model. Thirdly, the effectiveness of the cluster-based model TSR is greatly reduced in attack patterns with camouflage strategies. Finally, the effectiveness of the AMT method based on satisfaction evaluation is relatively low due to the existence of fake feedback.

### 4.4. Experiment 3: Validity of the Reverse Mechanism

In this experiment, we verify the effectiveness of the reverse mechanism for identifying workers. By adjusting the camouflage probability *p* of CAR and CAW in two cases, where 20% and 40% are camouflaged users, we set the effective identification rate, which is the proportion of truth values and actual values, as the performance metric. Then, we analyze the effectiveness of the TruthTrust and Non-Reverse model in identifying malicious users.

**Figure 9:**
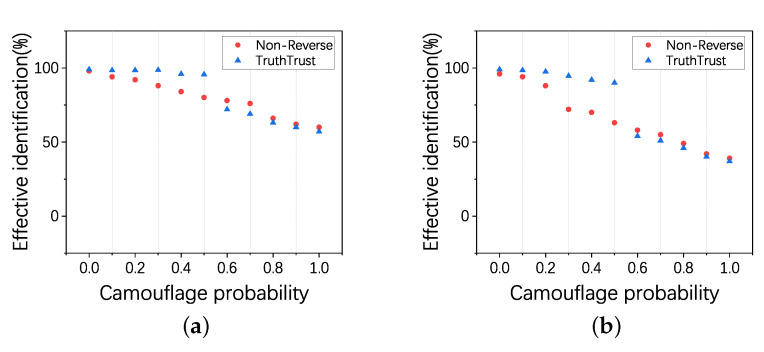
(**a**) shows that, with the change of the camouflage probability *p*, the effective identification of TruthTrust and Non-Reverse models under the proportion of CAR & CAW is 20%; (**b**) shows that, with the change of the camouflage probability *p*, the effective identification of TruthTrust and Non-Reverse models under the proportion of CAR & CAW is 40%.

From Figure 9a,b, we can observe that, when the probability of camouflage *p* is less than 0.5, the averaged effective identifications of the TruthTrust are 98.67% and 96.46%, higher than 84.2% and 63.4% achieved by Non-Reverse. However, when the probability is greater than 0.5, the effectiveness of TruthTrust is lower than Non-Reverse. In the case of low camouflage, the introduction of the reverse mechanism makes the feedback close to the real situation with high probability, however, when the probability of camouflage is high, although the feedback of shadow HITs are corrected, at the same time, the evaluations of normal workers will be incorrect. In general, the reverse mechanism of the TruthTrust has a good performance when the probability of camouflage is less than 0.5. However, when the probability is high, the reverse mechanism will make errors in the evaluation of good performers. However, the probability of camouflage in actual crowdsourcing generally does not exceed 0.5, so the reverse mechanism is suitable for most attack scenarios.

## 5. Conclusions

In this paper, we have presented a threat defense model based on the truth inference framework on a crowdsourcing platform. In the TruthTrust, we have analyzed three threat patterns and devised a new mechanism to evaluate the trustworthiness of workers and requesters. We established a trust-based recommendation mechanism according to interactive experience, distinguishing the recommended credibility and service quality evaluation. We proposed a reverse method to optimize the truth inference framework. The results of our experiments have demonstrated that the TruthTrust significantly outperforms CRH, TSR, and AMT, three traditional approaches, in both selecting honest workers and filtering out spam workers. In addition, we also proved the effectiveness of the reverse mechanism we proposed. However, when the proportion of malicious user exceeds 50%, the validity will be reduced because of the characteristics of the CRH framework. At the same time, when the probability of camouflage users is high, the effectiveness of the reverse mechanism will also be affected, so, in our future work, we will try to use machine learning clustering methods to reduce the weight of magnanimous malicious requesters, and we plan to extend the TruthTrust to defend more complex threat patterns in crowdsourcing.

## Figures and Tables

**Table 1:** Platform settings.

Items	Entries	Descriptions
Platform	Workers Kind of Tasks # of skills of each worker # of requesters # of HITs for training # of HITs for each round # of simulation cycles in one experiment # of experiments results are averaged HIT’s distribution	1000 30 5 80 5000 3000 20 5 Normal distribution

**Table 2:** Different identities of workers and requesters.

Identities	Descriptions
Spam Worker (SW) Camouflage Worker (CAW) Honest Worker (HW)	Always provide malicious service Provide a effective service with the probability *p* Always provide effective services
Spam Requester (SR) Camouflage Requester (CAR) Honester Requester (HR)	Always provide fake feedback Provide real feedback with the probability *p* Provide real feedback with 95% probability

**Table 3:** Threat patterns and the proportion of malicious users.

Threat Pattern	(SW%, CAW%)	(SR%, CAR%)
Threat Pattern A	(10,0), (20,0), ..., (50,0)	(10,0), (20,0), ..., (50,0)
Threat Pattern B	(10,0), (20,0), ..., (50,0)	(5,5), (10,10), (15,15), (20,20), (25,25)
Threat Pattern C	((5,5), (10,10), (15,15), (20,20), (25,25)	(5,5), (10,10), (15,15), (20,20), (25,25)

## Data Availability

The datasets generated during the current study are available from the corresponding authors on reasonable request.
